# Assistance of next-generation sequencing for diagnosis of disseminated Bacillus Calmette-Guerin disease with X-SCID in an infant: a case report and literature review

**DOI:** 10.3389/fcimb.2024.1341236

**Published:** 2024-02-12

**Authors:** Haiyang Zhang, Yi Liao, Zhensheng Zhu, Hanmin Liu, Deyuan Li, Sisi Wang

**Affiliations:** ^1^ Department of Pediatrics, West China Second University Hospital, Sichuan University, Chengdu, China; ^2^ Key Laboratory of Birth Defects and Related Diseases of Women and Children, Sichuan University, Ministry of Education, Chengdu, China; ^3^ Department of Radiology, West China Second University Hospital, Sichuan University, Chengdu, China; ^4^ Depertment of Bioinformation, Hugobiotech Co., Ltd., Beijing, China

**Keywords:** Bacillus Calmette-Guérin, next-generation sequencing, whole exome sequencing, immunodeficiency, vaccine

## Abstract

*Bacille Calmette-Guérin* (BCG) is a live strain of *Mycobacteriu*m *bovis* (*M.bovis*) for use as an attenuated vaccine to prevent *tuberculosis* (TB) infection, while it could also lead to an infection in immunodeficient patients. *M.bovis* could infect patients with immunodeficiency via BCG vaccination. Disseminated BCG disease (BCGosis) is extremely rare and has a high mortality rate. This article presents a case of a 3-month-old patient with disseminated BCG infection who was initially diagnosed with hemophagocytic syndrome (HPS) and eventually found to have X-linked severe combined immunodeficiency (X-SCID). *M.bovis* and its drug resistance genes were identified by metagenomics next-generation sequencing (mNGS) combined with targeted next-generation sequencing (tNGS) in blood and cerebrospinal fluid. Whole exome sequencing (WES) revealed a pathogenic variant in the common γ-chain gene (*IL2RG*), confirming X-SCID. Finally, antituberculosis therapy and umbilical cord blood transplantation were given to the patient. He was successfully cured of BCGosis, and his immune function was restored. The mNGS combined with the tNGS provided effective methods for diagnosing rare BCG infections in children. Their combined application significantly improved the sensitivity and specificity of the detection of *M.bovis*.

## Introduction

*Bacille Calmette-Guérin* (BCG) is a live strain of *Mycobacteriu*m *bovis* (*M.bovis*) for use as an attenuated vaccine to prevent *tuberculosis* (TB) infection. It remains the only vaccine against TB in general use in China. Localized adverse reactions, including hypersensitivity, abscess formation, and regional lymphadenitis following BCG vaccination, are common and self-limiting. Disseminated BCG disease (BCGosis) is extremely rare, while it can lead to high mortality in infants with the immunodeficient disease (Amanati et al., 2017; Lange et al., 2022). The early symptoms of this infection are very insidious and not specific, so a timely diagnosis could contribute to early treatment and be life-saving. High-throughput sequencing technology may have potential advantages in the diagnostic field of BCGosis. Metagenomics next-generation sequencing (mNGS) can detect a wide variety of organisms, but cannot achieve comprehensive detection of drug resistance genes. Targeted next-generation sequencing (tNGS) has a higher specificity than mNGS, and the species and origin of the bacteria can often be specifically identified. Through specific capture techniques, different drug resistance genes can be captured by tNGS (Beviere et al., 2023). Here, we report a rare case of severe X-linked severe combined immunodeficiency (X-SCID) with disseminated BCG infection that was initially presented with hemophagocytic syndrome (HPS) and eventually received umbilical cord blood transplant (UCBT). We identified the presence of infection quickly and found its resistance gene through mNGS combined with tNGS. Through this case, we proposed that mNGS combined with tNGS could effectively and rapidly identify BCGosis in potentially immunodeficient patients.

## Case presentation

A 3-month-old male infant had sudden, unprovoked bouts of high fever in October 2022. Anti-infective treatment with ceftriaxone for 3 days was ineffective. Miliary red papules appeared all over the body gradually He was transferred to the pediatric intensive care unit (PICU) of West China Second University on October 14, 2022. The patient did not have diarrhea. He had no history of recurrent infection since birth. His parents denied a family history of *Mycobacterium tuberculosis* (MTB) infection, immunodeficiency, or consanguineous marriage. There’s no history of sudden death in infancy in his immediate family. He was born at term and received an intradermal injection of 0.1 ml of BCG vaccine on the left upper arm on day 1 after birth. The growth and development of the child were within the normal range.

The physical examination at admission showed temperature of 38.6°C, respiratory rate of 56 times/min, pulse rate of 190 times/min, blood pressure of 73/59 mmHg, height of 60 cm, and weight of 6.8 kg. Scattered red papules were observed on the pale skin of his chest and abdomen. The BCG scar had crusted over without redness or suppuration. No thrush was found in the buccal mucosa. No enlarged superficial lymph nodes were palpable. The three-concave sign was positive, and the breath sounds of both lungs were rough without moist rales. An abdominal examination revealed hepatosplenomegaly. The liver was enlarged, with its lower edge 6 cm below the right costal margin. A palpable splenic edge was felt 8 cm below the left costal margin. Laboratory studies at the time of admission revealed hemoglobin (Hb) of 87 g/L, white blood cell count (WBC) of 4.7×10^9^/L, lymphocyte of 1.44×10^9^/L, neutrophils of 3.21×10^9^/L, and platelet count of 58×10^9^/L. CRP was 148.8 mg/L. PCT was 2.05 ng/ml. Coagulation tests suggested a hypercoagulable state (D-dimer was 27.95 mg/L, fibrin degradation products were 76.6 ug/L). The polymerase chain reaction (PCR) of Epstein-Barr virus (EBV) and Cytomegalovirus (CMV) was negative. The purified protein derivative (PPD) skin test (72 h) and T-cell spot test for tuberculosis infection (T-SPOT) were negative. A chest X-ray revealed slight infiltrate in both lungs. His initial diagnosis at admission was sepsis, severe pneumonia, and coagulopathy.

**Figure 1:**
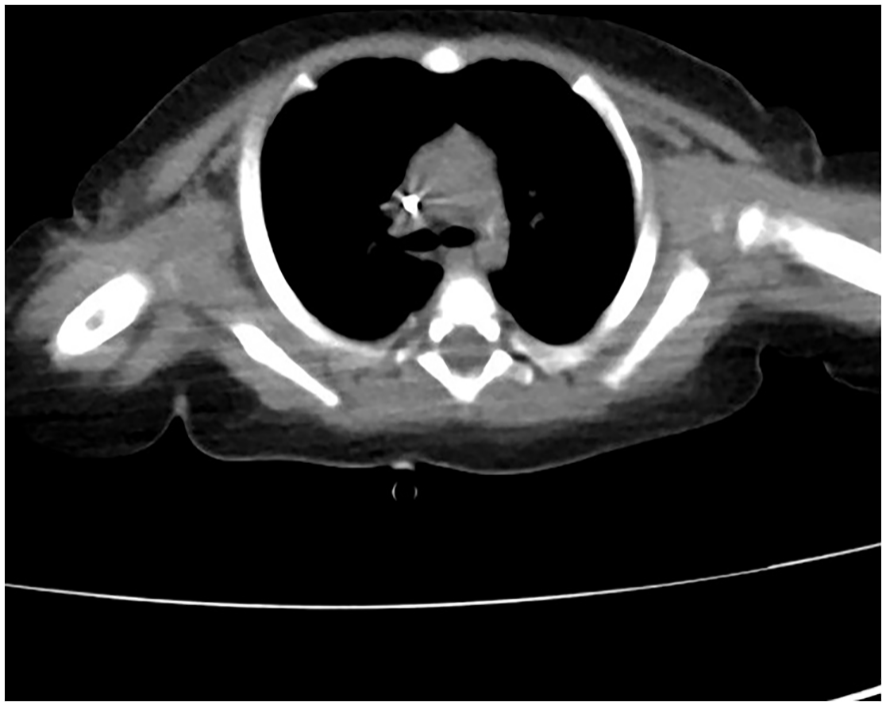
**The chest CT revealed the absence of a thymus**.

The patient received nasal high-flow ventilation immediately after admission. Anticoagulant therapy and empirical antibiotic treatment with intravenous imipenem (120 mg/kg/day, q6h) were administered to him. On the 2nd day of admission, the inflammatory markers were further increased (PCT > 150 ng/ml). The level of ferritin in the serum was 3022.70 ng/ml (normal range: 10-291 ng/ml). Dexamethasone (0.5 mg/kg/day, qd) was given as an empirical treatment for hemophagocytic syndrome (HPS). At the same time, the patient developed irritability and repeated moaning, indicating mild disturbance of consciousness. Subsequently, the patient began to show positive signs of meningeal irritation including projectile vomiting, increased muscle tone, and positive neck stiffness. We performed a cerebrospinal fluid (CSF) test because of his severe infection and worsening state of consciousness. The cytological and biochemical results of the CSF were normal. In addition, we applied samples of peripheral blood and CSF (2 ml each) for mNGS. On day 3, the results of natural killer (NK) cell activity were shown as 24.25%, and the soluble CD25 (sCD25) was 14784 pg/ml. The criteria of the HPS diagnostic criteria were fulfilled (Henter et al., 2007). HPS was confirmed. Etoposide (150 mg/m^2^/day, biw) was implemented with reference to the hemophagocytic lymphohistiocytosis-1994 protocol (Henter et al., 1997). On day 4, Xpert Mycobacterium tuberculosis and rifampicin resistance detection (Xpert MTB/RIF) from the sputum revealed positive for MTB, but negative for RIF. MTB-PCR was weakly positive in sputum. For this patient, we collected the deep sputum samples by nasotracheal suction. MTB complex (23178 reads in blood and 15710 reads in CSF) was detected via mNGS (Hugobiotech, Beijing, China, **Table 1**). A four-tuberculostatic drug regimen included isoniazid (H, 15 mg/kg/day, peros), rifampin (R, 15 mg/kg/day, peros), pyrazinamide(Z, 33 mg/kg/day, peros) and ethambutol (E, 19 mg/kg/day, peros). On day 7, *M.bovis* with resistance to pyrazinamide was identified by tNGS of the blood (Hugobiotech, Beijing, China). The drug resistance gene was *pncA* (**Table 1**). Levofloxacin(L, 15 mg/kg/day, peros) was added for anti-tuberculosis treatment, and pyrazinamide was discontinued. Immunologic studies showed T cells and NK cells were very low, while B cells were present. The immunoglobulin profile was within the lower range (**Table 2**). The chest computed tomography (CT) revealed the absence of a thymus and showed a hazy opacity (**Figure 1**). To sum up, the infant developed severe BCGosis and progressed rapidly, even developing complicated HPS. People with normal immune systems are usually not susceptible to *M.bovis*. The clinical findings, the imaging absence of thymus, and decreased cellular and humoral immune function suggested that the child may have primary immunodeficiency (PID). The clinical standard for diagnosis of PID was to conduct high-throughput sequencing of the whole genome exon. Therefore, 2 mL of peripheral blood from each of the family members was identified for a gene mutation through whole exome sequencing (WES). The results of the WES revealed a pathogenic variant (Exon 3: c.391C > T; p.Gln131Ter, acquired from the mother), in the common γ-chain gene (*IL2RG*), confirming X-SCID. This variant was a nonsense hemizygous variant, which caused the Glutamine at 131th changed the stop codon. The variant was not collected in healthy population database. Previous study reported this variant in association with X-linked SCID, it was predicted to cause loss of normal protein function either through protein truncation or nonsense-mediated mRNA decay. So, finally it was classified as pathogenic. In this case, the T cell counts were less than 0.05×10^9^/L, and the pathogenic gene was identified. According to the 2022 diagnostic criteria from the Primary Immune Deficiency Treatment Consortium’s (PIDTC’s) (Dvorak et al., 2023), we identified the case as a classical SCID.

**Table 1:** The data of mNGS and tNGS.

Method	Sample	Pathogen	Sequence number (reads)	Confidence	Relative abundance (%)	Drug resistance gene
mNGS	CSF	MTB	15710	high	10.81	no
mNGS	blood	MTB	23178	high	99.55	no
tNGS	blood	*M. Bovis*	4459	high	no	*pncA*

**Table 2:** Immunological data before/after hematopoietic stem cell transplantation (humoral immunity and cellular immunity).

	Pre-transplant	Post-transplant		
		3 months	4 months	6 months
Humoral immunity: lymphocyte subsets (×10^9^/L)				
CD3+ cells	0.03	0.1	1.56	2.73
CD4+ cells	0.01	0.08	0.49	0.84
CD8+ cells	0.01	0.03	1.06	1.88
CD4/CD8	1	2.67	0.46	0.45
CD19+ cells	0.97	0.01	0.01	0.02
CD56+ cells (NK cells)	0.01	0.23	1.05	1.03
Cellular immunity: immunoglobulins (g/L)				
IgG	1.7	20.6	16.9	18.2
IgA	0.07	0.15	0.07	0.17
IgM	0.16	1.23	4.16	1.31

**Figure 2:**
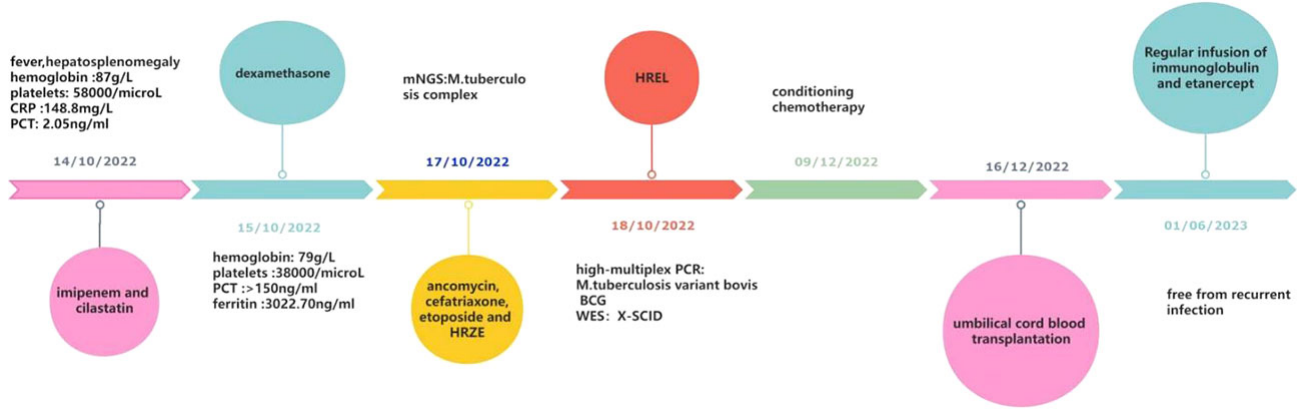
The treatment timeline of the patient.

1 month after the diagnosis of X-SCID, the patient received UCBT from unrelated donors (matched for 8/10 HLA alleles) after a conditioning chemotherapy regimen that included busulfan and cyclophosphamide. Regular infusions of immunoglobulin and etanercept were required in the post-transplant course. The HREL-based anti-tuberculosis regimen was used for 9 months. 6 months after UCBT, the patient was free from recurrent infections. During follow-up, there were no abnormalities in blood routine or ferritin. Re-examination of immunologic studies showed T cell counts (CD3, CD4 and CD8) and NK cell counts (CD56) returned to normal, while B cell counts (CD19) remained low (**Table 2**). After UCBT, we quantitatively assessed the post-transplant mosaicism status of this patient at regular intervals over 8 months. The final assessment results showed that donor cells accounted for 100% and T cells accounted for 99.82% of the peripheral blood after transplantation, both of which showed complete chimerism (**Table 3**). Mosaicism status was defined according to the rate of donor chimerism (DC) after transplantation. DC≥95% indicated a complete chimeric state (Locatelli et al., 2013). We have summarized the timeline of patient treatment and disease progression in **Figure 2**.

**Table 3:** The post-transplant rate of DC.

Detection time (Post-transplant)	The rate of DC (%)	
	Peripheral blood	T cell
2 weeks	99.36	93.12
4 weeks	70.1	81.6
2 months	85.95	89.16
3 months	95.86	95.67
4 months	98.42	99.82
5 months	100	99.25
6 months	99.85	99.63
8 months	100	99.82

### mNGS and tNGS methods

**Figure 3:**
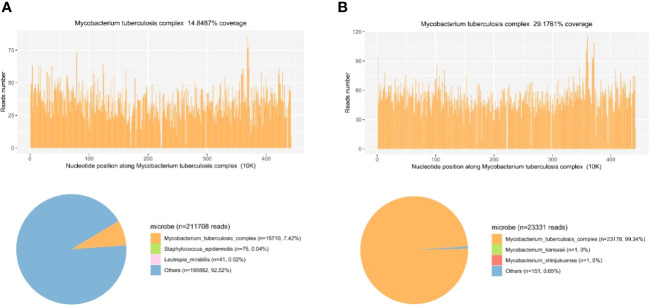
Diagnosis of MTB infection using mNGS. **(A)** The reads mapped to the MTB genome with coverage of 14.8487% in the blood. The distribution of species-specific reads aligns with the MTB in the blood. **(B)** The reads mapped to the MTB genome with coverage of 29.1761% in the CSF. The distribution of species-specific reads aligns with the MTB in the CSF.

The patient presented with sepsis at the beginning of admission. Pathogen cultures were performed on blood, sputum and CSF samples from the patient. All samples were cultured for 14 days, but the results were negative. In order to find the agent of infection as soon as possible, we tested the patient for mNGS. Blood and CSF samples were transported to Hugobiotech Co., Ltd. (Beijing) for nucleic acid extraction and mNGS. Blood samples (2-4 ml from the patient) were collected in the Cell-Free DNA BCT STRECK and then stored or shipped between 6-35°C for mNGS detection immediately. CSF samples (2-3 mL) were collected, then sterile sealed, stored at -20°C, and transported on dry ice for mNGS detection immediately. We extracted and purified DNA from 200 ul of plasma according to the instructions (YGZZ015, Hugobiotech, Beijing, China) for the QlAamp DNA Micro Kit (50) #56304. Qubit 3.0 fluorimeter (Q33216, Invitrogen, USA) and agar-gel electrophoresis (UVC1-1100, Major Science, USA) were performed to verify DNA concentration and quality. QIAseqTM Ultralow Input Library Kit was used to construct the DNA libraries for mNGS. We pooled qualified libraries with different barcode labeling and sequenced them using the Illumina Nextseq 550 sequencing platform (Illumina, San Diego, USA) and SE75bp sequencing strategy. By removing adapters, low-quality, low-complexity, short reads, and adapter-related data from mNGS sequencing data, high-quality data were obtained. We removed human reads by mapping them to the human reference genome using SNAP software and aligned the remaining reads to the Burrows-Wheeler Alignment (BWA) database. The National Center of Biotechnology Information (NCBI) provided the genomes for the database. The microbial composition of the sample was finally determined. The reads mapped to the MTB genome with coverage of 14.8487% in the blood (**Figure 3A**) and 29.1761% in the CSF (**Figure 3B**). The distribution of species-specific reads aligns with the MTB in the blood and CSF, respectively. The average length of each read was 75 bp.

After MTB was found in mNGS in both blood and CSF, tNGS were further performed for patients in order to screen out the species, subgroups and drug resistance genes of MTB. The DNA library for tNGS was constructed according to the operation manual of the General Kit for Identification and Drug Resistance Gene Detection of MTB Complex Group (YGZZ016, Hugobiotech, Beijing, China). Library quality control was performed with a Qubit 3.0 fluorimeter (Invitrogen, Q33216) and an Agilent 2100 Bioanalyzer (Agilent Technologies, Palo Alto, USA). Qualified DNA libraries with different barcodes were pooled and sequenced using the Illumina Nextseq 550 sequencing platform and SE75bp sequencing strategy. After the sequencing data was disassembled, the splices, low-quality simple repeats, and N-sequence data were removed to obtain high-quality sequencing data, and the human genome data was filtered through the BWA. The remaining sequencing data was compared with the dedicated microbial database blast to complete the species identification analysis of the target pathogen. The database was originally from NCBI. After manual collection and processing, it contained 49 kinds of microorganisms, including 10 types of MTB complex group and 39 kinds of nontuberculous mycobacteria (NTM). Blastn was used to compare the fragments with resistance markers to the reference sequences of drug resistance genes to find polymorphic loci (SNPS) and obtain relevant information on the corresponding drug resistance genes. Through the above tNGS detection process, we accurately identified the *M. Bovis.* The drug resistance gene was pncA.

We used sterile deionized water as a negative template control (NTC) and synthesized fragments with known quantities as a positive template control (PC). As quality control steps, NTC and PC were included in each wet lab procedure and bioinformatics analysis. In the case of *Cryptococcus* and MTB, positive mNGS results were considered when at least 1 unique read was mapped to species level and absent in NTC or when the ratio of reads per million (RPM) between sample and NTC (RPM_sample_/RPM_NTC_) > 5 as RPM_NTC_≠0. The above process ensured that mNGS combined with tNGS had significant sensitivity and specificity in detecting MTB.

### WES methods

Exons cover most of the genetic information related to protein coding, which plays a vital role in the normal function and health of life. WES generally has high sensitivity and can detect many types of mutations, including single nucleotide variations (SNVs) and small insertions or deletions (Indels). In this case, the genomic DNA obtained from all available family members was used for WES and Sanger Sequencing (Biosune). Then WES was performed based on the GenCap^®^ Whole Exon Gene Capture Probe V6.0. Multiple software programs were used to read the alignment to the human reference genome GRCh37/hg19 and annotate variants. The average coverage depth for the WES is 138.92 X, and the base coverage (≥20 X) in the target region is 97.01%. The identified mutations were assessed by referencing the Human Gene Mutation Database (HGMD), the Single Nucleotide Polymorphism Database (dbSNP), the Online Mendelian Inheritance in Man (OMIM), and ClinVar databases. Subsequently, all identified mutations were filtered based on clinical attributes, inherent patterns, and data from the Genome Aggregation Database (gnomAD) and the Exome Aggregation Consortium (ExAC) databases.

PCR samples were visualized on an agarose gel in the ABI PRISM 3730 genetic analyzer (Thermo Fisher Scientific, USA), which performed purification and sequencing using the terminated cycle sequencing method. The mutation sites were identified by comparing the DNA sequence with the NCBI database. A pair of primers were designed to amplify exon 3 of the *IL2RG* gene (NM_000206.3). The forward primer sequence was 5’-TACCTCCTCCCTTCCCATCA-3’. The reverse primer sequence was 5’-AAAGTCCAGAAGTGCAGCCA-3’, and the amplified fragment length was 298 bp. Purified PCR products with a size of 298 bp were sequenced by Sanger Sequencing. DNASTAR (Madison) software was used to analyze the sequencing results.

**Figure 4:**
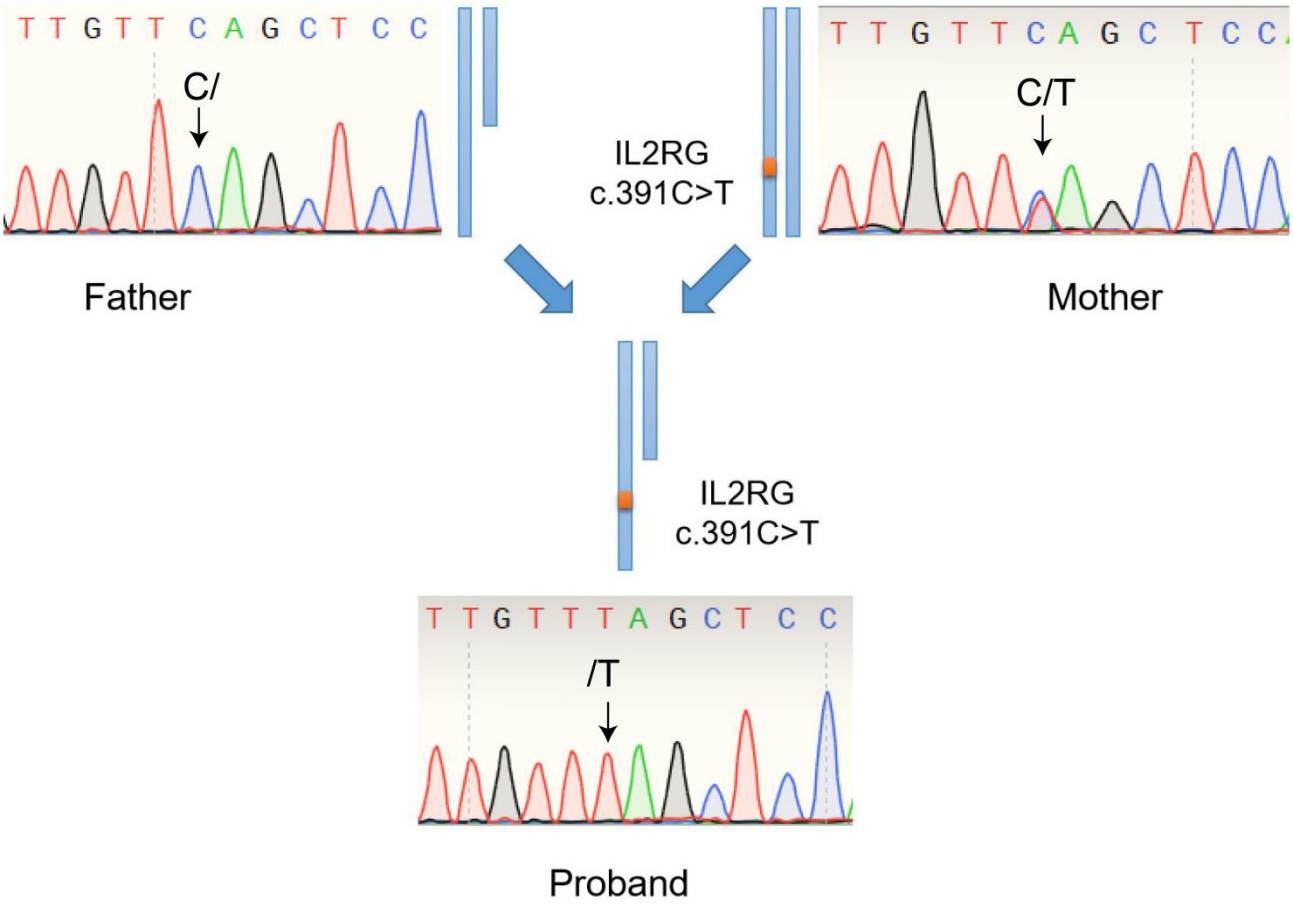
Genetic detection of the variant in the *IL2RG* gene (Exon 3: c.391C>T; p.Gln131Ter) among the patient, his father (wild-type) and his mother (carrier).

Finally, a flash analysis platform was used to analyze the pathogenicity of variation, and the possible variation loci were determined. The pathogenicity of variation loci was also analyzed according to ACMG (American College of Medical Genetics and Genomics) genetic variation classification criteria and guidelines (Richards et al., 2015). Considering the proband’s clinical features, SCID associated genes was searched and obtained from OMIM database (https://www.omim.org/). All variants located in exon and classic splicing regions was obtained for further analysis. Variants with a minor allele frequency of <0.01 in the gnomAD/ExAC database were obtained for following pathogenic classification. The IL-12/23/ISG15-IFN-γ and other related genes associating with X-linked SCID were carefully considered. Through the above WES and Sanger Sequencing process, we accurately identified the mutation in the *IL2RG* gene (Exon 3: c.391C>T; p.Gln131Ter) in the patient (**Figure 4**). The genetic detection of the father was wild-type, and the genetic detection of the mother was with respect to the mutation in the *IL2RG* gene (Exon 3: c.391C>T; p.Gln131Ter. **Figure 4**).

### Systematic review

We searched PubMed, Web of Science databases, Embase, and Medline from 1993 to 2023. The keywords were “X-SCID” and “BCG disease”. We extracted the following details from each article: first author, vaccination, clinical manifestation, detection method, complication, therapy and outcome. We summarized a total of 7 articles from our literature search in **Table 4** (Culic et al., 2004; Huang et al., 2006; Jaing et al., 2006; Bacalhau et al., 2011; Shi et al., 2020; Maron et al., 2021; Liu et al., 2022).

**Table 4:** BCG disease in X-SCID patients in recent 30 years.

Author	Age	BCG vaccination	Clinical manifestation	Screening for TB	Chest imaging	Diagnostic methods	Drug resistance	Complicated with HPS	Antituberculosis drugs	Immune reconstitution	Outcome
Liu S (Richards et al., 2015)	5m	vaccinated at birth	fever, cough and respiratory insufficiency	not mentioned	absence of athymus, opacities	PCR	no	no	H、R、E、Lfx	HSCT	alive
Maron G (Liu et al., 2022)	4m	vaccinated at birth	fever,cough and papule	tuberculosis antibody(-)	infectious lesions	PCR	no	yes	H, R, Z	no	death
Shi B (Maron et al., 2021)	4m	vaccinated at birth	fever and lymphadenopathy	acid fast bacilli stain (-)	not mentioned	PCR	no	no	H, R, E, Lfx	gene therapy	alive
Bacalhau S (Shi et al., 2020)	4m	vaccinated at birth	fever and pustules	not mentioned	not mentioned	not mentioned	no	yes	Lzd, Mpm, H, R, E, Clr	HSCT	alive
Jaing TH (Bacalhau et al., 2011)	5m	vaccinated at the third day of life	decreased appetite and tachypneic	not mentioned	infectious lesions	sputum culture	no	no	H, R, E, Cfx	UCBT	alive
Huang LH (Jaing et al., 2006)	8m	vaccinated at the third day of life	fever and rash	not mentioned	infectious lesions and absence of athymus	PCR	no	no	H, R, Z	no	death
Culic S (Huang et al., 2006)	6m	not mentioned	chronic diarrhea	not mentioned	not mentioned	not mentioned	no	no	not mentioned	no	death

## Discussion

The BCG is a live attenuated vaccine strain of *M.bovis* that is commonly used to prevent MTB infection. Once BCG is inoculated into the body, it can cause an extremely mild asymptomatic infection, thereby inducing the body to produce memory T lymphocytes and ultimately achieving the purpose of preventing tuberculous meningitis and disseminated MTB infection in infants and young children. The BCG vaccination is considered safe for competently immune infants. However, in patients with immunosuppression or BCG treatment for bladder cancer, postvaccination or posttreatment adverse reactions may occur, which may manifest as local adverse reactions, lymphadenitis, disseminated BCG disease, immune reconstructive inflammatory syndrome, and osteomyelitis (Toskas et al., 2022). *M.bovis* is naturally resistant to pyrazinamide (de la Morena et al., 2017). From a multicentre study, the incidence of BCGosis and BCGtis in SCID was 34% and 19%, respectively (Marciano et al., 2014). The overall mortality of BCGosis ranged from 50% to 71% (Amanati et al., 2017; Ong et al., 2020). While a potentially life-threatening BCGosis with an overall fatality rate of 50% and higher could arise in PID, which was 0.06 to 1.56 per million vaccinated children (Amanati et al., 2017; Lange et al., 2022). Early detection and appropriate treatment are lifesaving for them. However, it often took 1.5 to 2 months from the early onset to the diagnosis of MTB infection, probably due to a lack of exposure and poor specificity of symptoms in children (Ong et al., 2020; Thomas et al., 2022).

In this case, The infant was predisposed to *M.bovis* due to SCID. The known gene defects that cause SCID include *IL2RG, ADA, IL7R, RAG1, RAG2, JAK3, DCLRE1C, PTPRC, BCL11B, RMRP, CD3E, CD247, NHEJ1, CORO1A, LIG4, PRKDC, LAT, RAC2, AK2* and *TTC7A* (Tangye et al., 2020). Considering the proband’s clinical features, SCID associated genes was obtained from OMIM database (https://www.omim.org/). The IL-12/23/ISG15-IFN-γ and other related genes associating with SCID were carefully considered. All variants located in exon and classic splicing regions was obtained for further analysis. Variants with a minor allele frequency of <0.01 in the gnomAD/ExAC database were obtained for following pathogenic classification according to the ACMG guideline. Finally, the proband was diagnosed with X-SCID by WES. PID is a group of heterogeneous diseases dominated by T/B cell defects accompanied by other cell defects to varying degrees. X-SCID is due to defects in the *IL2RG*. IL2 was discovered as a T cell growth factor that can potently boost the cytolytic activity of NK cells. This receptor subunit is shared by several different cytokine receptor complexes. Pathogenic variants in this gene often cause absent T cells and NK cells with nonfunctional B cells via the blockade of multiple cytokines (Spolski et al., 2018). X-SCID usually manifests as extreme susceptibility to infections, which may lead to death in the first few months of life unless immunologic reconstitution is achieved (Lange et al., 2022). For patients with X-SCID, the tubercle bacilli continue to proliferate, even leading to BCGosis after the BCG vaccine due to an ineffective cell-mediated immune response. In China, BCG is regularly recommended for newborns in the first month of their lives without screening for PID. In the majority of immunocompromised populations, BCG might be a major cause of infection and an obstacle to future immune reconstitution (Zhang et al., 2011; Norouzi et al., 2012). Further screening should be performed for children with repeated infections, even if the screening results for latent TB infection are negative. Children with X-SCID had the highest survival rate after receiving transplantation before 3.5 months (Hardin et al., 2022). Key clinical information on the incidence, prevalence, treatment status and long-term prognosis of X-SCID in China is still incomplete. Due to the large population base and high testing costs, neonatal screening for X-SCID is not widespread in China. X-SCID network registration and data collection for clinical epidemiology are further needed in China. For infants with a family history, immunological screening should be performed before BCG vaccination. The family histories included a history of immunodeficiency in the immediate family or an unexplained early death. If the parents are recently married, the child should also be included in the scope of PID screening. The ultimate cure for X-SCID is suggested to be hematopoietic stem cell transplantation. After transplantation, regular monitoring of humoral immunity, cellular immunity and mosaicism status is required to comprehensively evaluate the transplantation effect and immune reconstitution (Locatelli et al., 2013). Among them, the CD4/CD8 T cell ratio is an indispensable monitoring indicator. The reversed CD4/CD8 T cell ratio usually indicates immunosuppressive status or reduced rejection after transplantation (Kimura et al., 2020). In this case, both CD4 T cell count and CD8 T cell count returned to normal after UCBT, while the CD4/CD8 T cell ratio was inverted, suggesting that the risk of rejection was reduced in the process of immune reconstitution.

The patient’s initial clinical symptoms were consistent with HPS. Such atypical clinical symptoms also hinder early identification of MTB infection. HPS is an aggressive and fatal syndrome of excessive inflammation due to abnormal immune activation caused by the absence of normal downregulation by activated macrophages and lymphocytes. The occurrence of HPS leads to the production of cytokine abundance and an imbalance of the host immune response, which can cause multiple organ damage or failure (Allen and McClain, 2015). TB-associated HPS has been shown to have a poor prognosis and high mortality rates (up to 50%) (Padhi et al., 2015). Several cases of HPS due to BCG have been reported in adults with bladder cancer after intravesical instillation of BCG, but rarely in children (Bacalhau et al., 2011). In this case, BCG induced the polarization of M0-type macrophages into M1-type macrophages (Liu et al., 2015). Key transcription factors such as NFkB, STAT1, STAT5 and IRF3 can mediate M1-type macrophages’ release of numerous cytokines and chemokines (Bosco, 2019). The lack of normal feedback regulation leads to excessive macrophage polarization with highly elevated levels of cytokines, which triggers the predisposing factors of HPS. In addition, this patient had a severe congenital immune deficiency, which not only predisposed him to severe invasive infections but also induced a systemic cytokine storm. As soon as the diagnosis of HPS is suspected, treatment with etoposide and corticosteroids should be initiated (Henter et al., 2007). Nevertheless, these immunosuppressive treatments can exacerbate the course of the BCG infection. Thus, confirming the diagnosis of BCG infection and timely anti-tuberculosis treatment need to be synchronized and implemented as quickly as possible. Shi B has reported a pediatric case of HPS secondary to BCG disease in which the treatment ultimately failed due to a delayed diagnosis (Maron et al., 2021). Our strategy was to maintain a full-dose regimen of HPS and avoid neutropenia. In addition, we adopted the method of mNGS combined with tNGS to quickly identify *M.bovis* and its resistance genes, and developed a more targeted drug regimen as soon as possible. At the same time, levofloxacin, a second-line anti-tuberculosis drug, was added in time to achieve full coverage of MTB.

We reviewed the literature reporting BCG infection in X-SCID patients over the past thirty years. All seven children presented with fever, cough, chronic diarrhea, or other nonspecific manifestations. All reported cases began with clinical symptoms in infancy. Screening results for latent TB infection were negative. Two of the children had male relatives who died from infantile infections. After anti-tuberculosis therapy and undergoing transplantation or gene therapy, four patients had achieved immunologic reconstitution. Unlike our case, which used mNGS combined with tNGS to detect *M.bovis* and found its drug resistance, most of the MTB reported in these literatures were detected by PCR, and no drug resistance genes were found (Culic et al., 2004; Huang et al., 2006; Jaing et al., 2006; Bacalhau et al., 2011; Shi et al., 2020; Maron et al., 2021; Liu et al., 2022). The conventional etiological culture-based detection is time-consuming (generally 2–6 weeks) (Assemie et al., 2020). Positive culture specimens need a further drug sensitivity test, but it takes another 2 to 4 weeks. In recent years, the use of mNGS for the detection of MTB and NTM has received considerable attention because of its shortened turnaround time and significant sensitivity (Gu et al., 2019). mNGS does not need to refer to the culture results to analyze the results, nor does it require specific amplification. It can directly conduct non-differential and non-selective high-throughput sequencing of nucleic acids in clinical samples and detect pathogenic microorganisms (including viruses, bacteria, fungi, and parasites) promptly and objectively. Compared with traditional methods, the sensitivity and positive predictive value of mNGS in the diagnosis of MTB infection were significantly higher than those of smear, culture and GeneXpert MTB/RIF. MTB is an intracellular growth bacterium that releases fewer extracellular nucleic acids. MTB was considered positive by mNGS when at least 1 read was mapped, due to the difficulty of DNA extraction and low possibility of contamination (Miao et al., 2018). Due to the completely conserved DNA sequences in the MTB complex and insufficient nucleic acid sequence information, it is difficult for mNGS to determine the species within the MTB complex and detect multiple resistant mutations (Chae and Shin, 2018). tNGS technology is a targeted enrichment sequencing technology based on NGS, which combines multiple PCR amplifications with sequencing technology. It has a clear range of target pathogens. The subspecies and subtypes can be further differentiated. This method contributes to the early diagnosis of BCG disease. In addition, it can capture numerous different drug resistance gene sequences, which enable molecular diagnosis of drug resistance (Murphy et al., 2023). Compared with mNGS, tNGS has the remarkable advantages of a clear pathogen spectrum and a low sequencing cost. We adopted mNGS combined with tNGS for species identification. This combination of the two methods provides rapid, sensitive, and specific identification of *M. bovis* and detects resistance to antituberculosis drugs. Furthermore, the combination of mNGS and tNGS is also useful for evolutionary tracking, strain typing, and virulence prediction of MTB.

It is extremely rare for HPS to be triggered by BCG. Both HPS and BCGosis have a high mortality rate, so early diagnosis of the cause and treatment are critical. The favorable sensitivity and specificity of mNGS combined with tNGS in the identification of *M.bovis* can help us identify specific pathogens and drug resistance. However, in the detection of susceptibility of MTB to various drugs, the genotype method cannot completely replace traditional phenotyping because the genetics of drug resistance in MTB are not completely clear (Ko et al., 2019). At present, there is relatively little literature on the value of high-throughput sequencing technology in the diagnosis of BCGosis. In this case report, we only show the experience of a single patient. While it provides in-depth information about this particular case, it’s not generalizable to a larger population. We also did not map a phylogenetic tree analysis of *M.bovis* based on existing NGS information. Relevant case-control studies and cohort studies can be designed to further explore the clinical significance of mNGS combined with tNGS in the future. The clinical significance of mNGS combined with tNGS needs to be further explored in the future. With the continuous optimization of high-throughput sequencing technology, the recognition of the value of high-throughput sequencing technology in the diagnosis of latent MTB infection, BCGosis and drug resistance will gradually deepen.

## Conclusion

In conclusion, we report the successful treatment of a BCGosis case. For PID, BCG vaccine injection may lead to BCGosis. This disease can lead to some non-specific complications, such as HPS. The mNGS combined with the tNGS may be a good choice to identify the *M.bovis* and its drug resistance in children. Their combined application significantly improved the sensitivity and specificity of the detection of *M.bovis*.

## Data availability statement

The datasets presented in this study can be found in online repositories. The names of the repository/repositories and accession number(s) can be found in the article/**Supplementary Material**.

## Ethics statement

The studies involving humans were approved by West China Second University Hospital of Sichuan University. The studies were conducted in accordance with the local legislation and institutional requirements. Written informed consent for participation in this study was provided by the participants’ legal guardians/next of kin. Written informed consent was obtained from the individual(s), and minor(s)’ legal guardian/next of kin, for the publication of any potentially identifiable images or data included in this article.

## Author contributions

HZ: Conceptualization, Data curation, Investigation, Resources, Writing – original draft. YL: Data curation, Methodology, Writing – review & editing. ZZ: Validation, Visualization, Writing – review & editing. HL: Formal Analysis, Project administration, Supervision, Writing – review & editing. DL: Data curation, Supervision, Writing – review & editing. SW: Conceptualization, Data curation, Methodology, Resources, Software, Writing – review & editing.
